# Utilizing pre-operative MR imaging and adapting optimal needle puncture approach to improve radiofrequency ablation fraction of thoracic dorsal root ganglia

**DOI:** 10.1038/s41598-021-98050-4

**Published:** 2021-09-20

**Authors:** Bing Li, Yayong Huang, Yong Zhang, Sushant Kumar Das, Chuan Zhang, Yang Li, Xiaoxue Xu, Hanfeng Yang, Yong Du

**Affiliations:** 1 The First Affiliated Hospital, Jinan University, Guangzhou, 510630 People’s Republic of China; 2 Department of Radiology, Affiliated Hospital of North Sichuan Medical College, 63 Wenhua Road, Nanchong, 637000 Sichuan People’s Republic of China; 3 Department of Radiology, Xuzhou City Center Hospital, 199 South Jiefang Road, Xuzhou, 221009 Jiangsu People’s Republic of China; 4 Department of Radiology, People’s Hospital of Deyang City, 173 North Taishan Road, Deyang, 618000 Sichuan People’s Republic of China

**Keywords:** Neuropathic pain, Chronic pain

## Abstract

This experimental study evaluates the location of thoracic dorsal root ganglions (DRGs) through magnetic resonance imaging (MRI) scans, and evaluates the radiofrequency ablation (RFA) fraction of different puncture approaches on distinct DRG locations. Eight normal adult corpse specimens were used as thoracic spine specimens. An MRI examination was performed on each specimen using the following MRI sequences: STIR T2WI, fs-FRFSE T2WI, and 3D FIESTA-c. Then thoracic spine specimens (n = 14) were divided into three groups for RFA: Group A, using a transforaminal approach irrespective of DRG location; Group B, using a transforaminal, trans-lateral-zygapophysial or translaminar approach according to the DRG location; and Group C using a combination of puncture approaches. The quality of visualization of thoracic DRGs on STIR T2WI, fs-FRFSE T2WI, and 3D FIESTA-c scans were 53.5% (77/144), 88.2% (127/144), and 93.1% (134/144), respectively. In group A, the RFA fractions of the extraforaminal DRGs (N = 29), intraforaminal DRGs (N = 12) and intraspinal DRGs (N = 7) via a transforaminal approach were 72.6 ± 18.9%, 54.2 ± 24.8% and 32.9 ± 28.1% respectively. In group B, RFA of extraforaminal DRGs via a transforaminal approach (N = 43) or a trans-lateral zygapophysial approach (N = 45) led to ablation fractions of 71.9 ± 15.2% and 72.0 ± 17.9%, respectively; RFA of intraforaminal DRGs via a transforaminal approach (N = 14) or a translaminar approach (N = 16) led to ablation fractions of 57.1 ± 18.0% and 52.5 ± 20.6%, respectively; RFA of intraspinal DRGs via a transforaminal approach (N = 12) or a translaminar approach (N = 14) led to ablation fractions of 34.8 ± 24.6% and 71.8 ± 16.0%, respectively. In group C, the combined approach led to an ablation fraction for extraforaminal DRGs (N = 69) of 82.5 ± 14.1%, for intraforaminal DRGs (N = 39) of 81.5 ± 11.8%, and for intraspinal DRGs (N = 36) of 80.8 ± 13.3%. MRI can accurately assess DRG location before RFA. Adopting different and combined puncturing approaches tailored to different DRG locations can significantly increase the DRG RFA fraction.

## Introduction

The dorsal root ganglion (DRG) is the primary afferent center of pain transmission and plays an important role in the pathogenesis of neuropathic pain (NP)^1^. If the sensory signal of the DRG is interrupted, the pain will be reduced or disappear. Thus, the DRG is an important target for NP treatment^2^.

**Figure 1:**
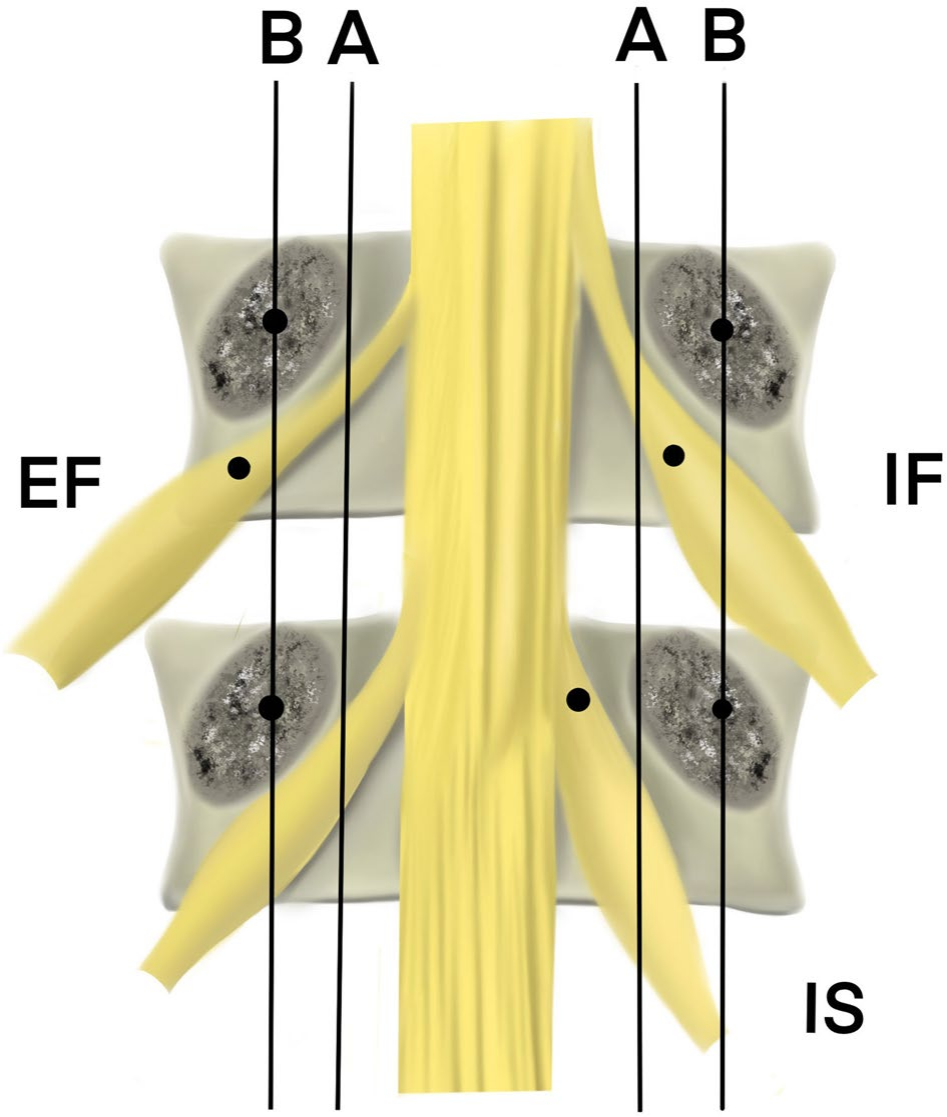
DRG locations. *IS* intraspinal, *IF* intraforaminal, *EF* extraforaminal. A is the line connecting the medial borders of pedicles of vertebral arch. B is the line connecting the centers of pedicles. If the proximal end of DRG lies proximal to A, it is intraspinal location; between A and B, intraforaminal; and distal to B, extraforaminal.

Nerve radiofrequency ablation (RFA) is a method of treating pain through the use of electrical current to generate thermal damage to the central and peripheral nervous systems, destroying nerve fibers and blocking the transmission of nerve impulses^3^. RFA of DRGs has been widely utilized for the treatment of various types of chronic pain^3–5^. Common forms of NP include trigeminal neuralgia (TN) and post herpetic neuralgia (PHN), but, interestingly, the clinical effectiveness of RFA for these two targets is significantly different. It has been reported that the RFA fraction of TN for treatment of trigeminal ganglia pain is about 90–96.3%^6^, however the RFA fraction of DRG for treatment of PHN is only about 25–55%^7^. After reviewing the relevant literature, we believe the lack of image localization of DRGs before surgery and insufficient research on the impact of DRG puncture pathways may be the key reasons for poor RFA fraction of DRG. Thoracic DRGs vary in position. Kikuchi et al. reported that DRGs could be classified into three types according to their location: intraspinal, intraforaminal, and extraforaminal (Fig. 1)^8^. At present, the transforaminal approach is the most widely used puncture approach for RFA of thoracic PHN, because this approach is less difficult to puncture and has a lower complication rate^9^. This nonspecific puncture approach may be one of the reasons for the poor RFA fraction of PHN.

Thus, it is clinically important to show the position of a DRG in order to determine the best approach(es) for cannula insertion in planning its for RFA. Magnetic resonance imaging (MRI) has great advantages in nerve root imaging, but presently, there are few MRI studies on thoracic DRG images. The present study evaluates the locations of thoracic DRG prior to RFA through MRI scans. Moreover, this study assesses the RFA fraction of different puncture approaches on varying locations of DRGs in order to improve the RFA fraction on thoracic DRGs.

## Results

### Quality of visualization of DRG with MRI

**Figure 2:**
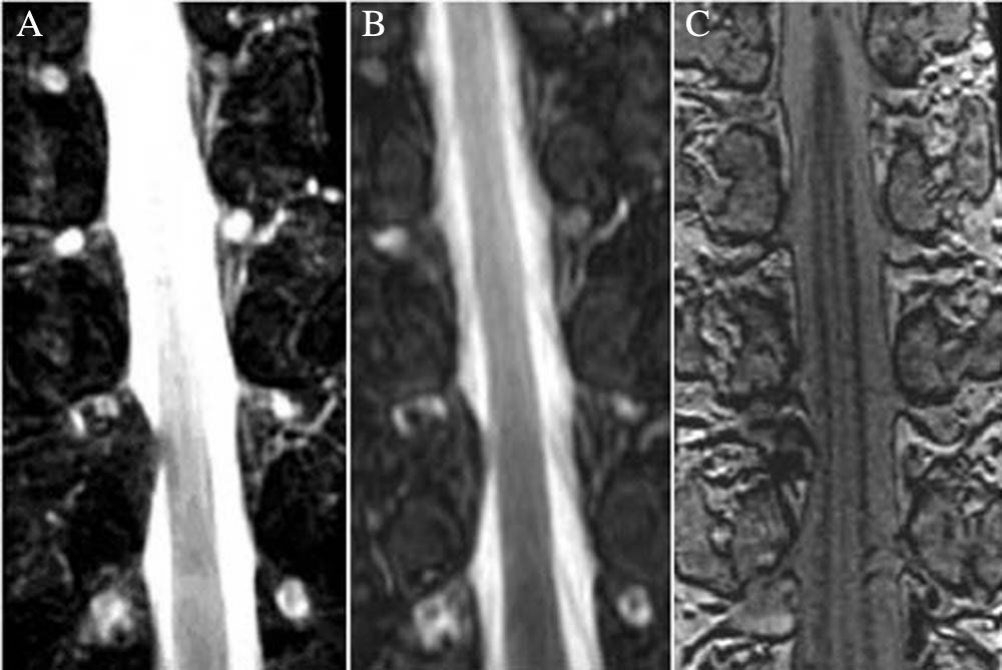
Different MRI sequences show DRG locations. (**A**) FSE-STIR T2WI; (**B**) Fs-FRFSE T2WI; (**C**) 3D FIESTA-c.

DRGs showed high-signal oval shape nodules on STIR and fs-FRFSE T2WI scans, and showed slightly higher signals on 3D FIESTA-c scans, with linear low-signals at the edges. The quality of visualization of thoracic DRGs on STIR T2WI, fs-FRFSE T2WI, and 3D FIESTA-c scans were 53.5% (77/144), 88.2% (127/144), and 93.1% (134/144), respectively (χ^2^ = 78.84, *p* < 0.05) (Table 1; Fig. 2). The number of DRGs in extraforaminal, intraforaminal, and intraspinal positions were 88, 30 and 26 respectively.

**Table 1:** MRI quality of visualization of DRG.

Sequence	T1–2	T3–6	T7–10	T11–12	Total	% of DRGs visible
FSE-STIR T2WI	0	21	34	22	77	53.5
fs-FRFSE T2WI	20	37	46	24	127	88.2
3D FIESTA-c	19	40	46	24	129	93.1.5

### RFA fraction

**Figure 3:**
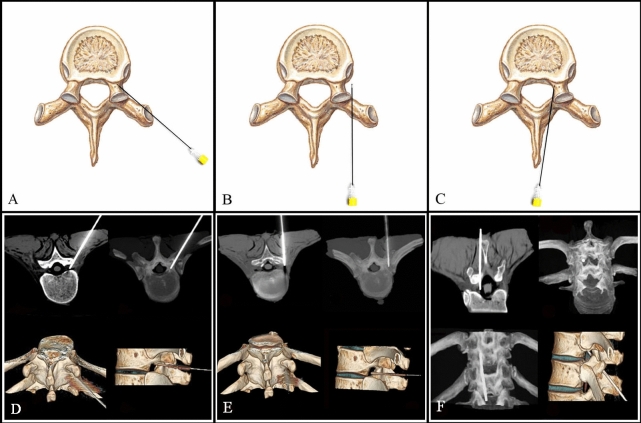
(**A**) Schematic of transforaminal approach; (**B**) Schematic of trans-lateral-zygapophysial approach; (**C**) Schematic of translaminar approach. (**D**) Puncture DRG via a transforaminal approach. CT 3D reconstruction shows position of the needle. (**E**) Puncture DRG via a trans-lateral-zygapophysial approach. (**F**) Puncture DRG via a translaminar approach.

**Figure 4:**
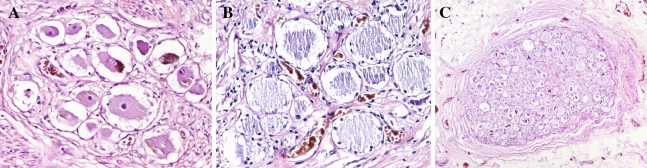
(**A**) (Magnification: 200 times). (**A**) Normal neurons in the ganglion, the cell nucleus is deeply stained, and the shape is normal; (**B**) (magnification: 200 times). After RFA, the neuron cell body produces coagulative necrosis, the nucleus dissipates, and the cell contour remains; (**C**) (magnification: 40 times). Shows the ratio of damaged neurons to the entire ganglion.

**Figure 5:**
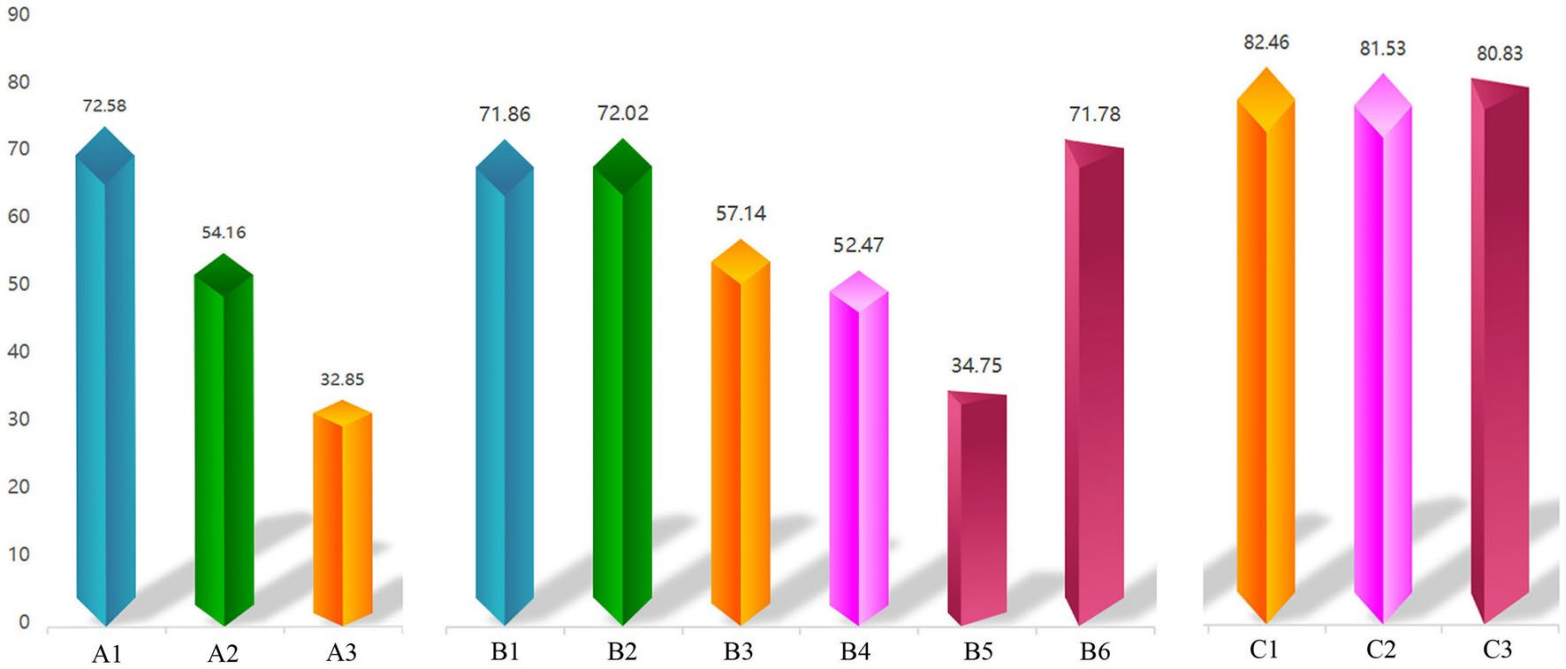
RFA fraction of each Subgroups. Subgroups name of A1–C3 refer to Table 3.

In group A, the RFA fractions of the extraforaminal DRGs (N = 29), intraforaminal DRGs (N = 12) and intraspinal DRGs (N = 7) via a transforaminal approach were 72.6 ± 18.9%, 54.2 ± 24.8% and 32.9 ± 28.1% respectively. In group B, RFA of extraforaminal DRGs via a transforaminal approach (N = 43) and a trans-lateral zygapophysial approach (N = 45) led to ablation fractions of 71.9 ± 15.2% and 72.0 ± 17.9%, respectively. RFA of intraforaminal DRGs via a transforaminal approach (N = 14) and a translaminar approach (N = 16) led to ablation fractions of 57.1 ± 18.0% and 52.5 ± 20.6%, respectively; RFA of intraspinal DRGs via a transforaminal approach (N = 12) and a translaminar approach (N = 14) led to ablation fractions of 34.8 ± 24.6% and 71.8 ± 16.0%, respectively. In group C, the combined approach led to an ablation fraction for extraforaminal DRGs (N = 69) of 82.5 ± 14.1%, for intraforaminal DRGs (N = 39) of 81.5 ± 11.8%, and for intraspinal DRGs (N = 36) of 80.8 ± 13.3% (Figs. 3, 4, 5; Table 2).

**Table 2:** RFA fraction of three groups.

Group	DRG position	Puncture approach	Total	RFA fraction (%)	*P* value (ANOVA)				
A	Extraforaminal	Transforaminal approach (A1)	29	72.6 ± 18.9	*		§		
	Intraforaminal	Transforaminal approach (A2)	12	54.2 ± 24.8	*		※		
	Intraspinal	Transforaminal approach (A3)	7	32.9 ± 28.1	*	&	†		
B	Extraforaminal	Transforaminal approach (B1)	43	71.9 ± 15.2				❄	
		Trans-lateral-zygapophysial approach (B2)	45	72.0 ± 17.9					✧
	Intraforaminal	Transforaminal approach (B3)	14	57.1 ± 18.0				✺	
		Translaminar approach (B4)	16	52.5 ± 20.6					✡
	Intraspinal	Transforaminal approach (B5)	12	34.8 ± 24.6	#			⋆	
		Translaminar approach (B6)	14	71.8 ± 16.0	#	&			✢
C	Extraforaminal	Transforaminal combined with trans-lateral-zygapophysial approach (C1)	69	82.5 ± 14.1			§	✺	✧
	Intraforaminal	Transforaminal combined with translaminar approach (C2)	39	81.5 ± 11.8			※	❄	✡
	Intraspinal	Transforaminal combined with translaminar approach (C3)	36	80.8 ± 13.3			†	⋆	✢

*P < 0.05 during comparison between DRG positions within Group A; # *P* < 0.05 during comparison between puncture approaches within DRG position subgroups in Group B; & *P* < 0.05 during comparison between puncture approaches within DRG position subgroups of Groups A and B; §,※,† *P* < 0.05 during comparison between puncture approaches within DRG position subgroups of Groups A and C; ❄, ✧, ✺, ✡, ⋆, ✢*P* < 0.05 during comparison between puncture approaches within DRG position subgroups of Groups B and C.

In group A, there were significant differences in the ablation fractions between the extraforaminal subgroup, the intraforaminal subgroup, and the intraspinal subgroup (*P* < 0.05). In group B, there were significant differences in ablation fractions between the extraforaminal and intraforaminal subgroups, and between the intraforaminal and intraspinal subgroups. There were also significant differences in ablation fractions between the three DRG locations of groups A and C, and groups B and C (*P* < 0.05). There were no significant differences between other DRG locations (Table 2).

## Discussion

In this study, the DRG imaging characteristics were obtained by MRI scans prior to ablation treatment, and ablation fractions achieved with different puncture approaches examined for different DRG locations. Combined puncture approaches were also employed for certain subgroups. Our results demonstrated that MRI scans can effectively evaluate DRG position and display anatomical information. The 3D FIESTA-c scan provided the best visualization of thoracic DRGs. In addition, we found different puncture approaches to specific DRG locations can significantly influence the achieved ablation fraction.

MRI is a multi-parameter imaging technology with high tissue resolution and has become the preferred method for neuroimaging research^10^. The value of MRI in thoracic DRG evaluation lies in the accurate assessment of the anatomy and location of the DRG prior to surgery, thereby providing a reliable reference for the clinical treatment of NP. Although the three MRI sequences used in this study had different quality of visualizations of the DRG, the anatomical information regarding the DRG location was consistent with all the MRI sequences.

T2-weighted STIR scans demonstrated the lowest quality of visualization of thoracic DRGs (53.5%), which may be related to low signal suppression selectivity, thicker layer depth, and artifacts caused by respiratory motion. The quality of visualization of thoracic DRGs by FRFSE T2WI scanning was 88.2%, with 3D-FIESTA-c having a slightly higher visualization quality (93.1%) illustrating the potential of both methods to depict thoracic DRGs. The 3D-FIESTA-c scans are better suited to use in with multi-planar volume reconstruction to observe the thoracic DRG, which clearly showed the anatomy and position distribution of the DRG, and could serve as a vital imaging sequence for interventional therapy under image guidance. This study is distinct from the studies by Grams et al., which were mainly focused on the anatomical morphology of lumbar spinal nerve and DRGs in coronal volume reconstruction images^11,12^.

3D-FIESTA belongs to the steady-state free precession imaging sequence, and simultaneously collects the fully re-focused steady-state sequence of the free induction decay (FID) pre-excitation signal (S−) and the excited signal (S+)^13^. The applied gradient are in a balanced state, and no phase shift occurs during the time of repetition (TR). The echo time (TE) used is only about 1/2 of TR, which is the steady-state imaging sequence with the fastest signal acquisition speed and the highest signal-to-noise ratio, which can form a strong T2 contrast helpful for showing the anatomical details of peripheral nerves^13^. 3D-FIESTA sequences generate high-resolution MR datasets, and images can be reconstructed in different planes with various section thicknesses^14^. This capability optimized the identification of the DRG. The present study showed that the DRG and its branches appeared as linear low signal, while the fatty tissue surrounding the DRG appeared as high signal structures on 3D-FIESTA images. The DRG could be easily identified in 3D-FIESTA images, which were considered better than STIR and other MRI sequences.

From our experimental results regarding RFA approach, it can be observed that although the ablation fraction of the extraforaminal DRGs was 72% using the transforaminal approach, the ablation fraction of intraforaminal and intraspinal DRGs using the transforaminal approach was only 54% and 32%, respectively. According to imaging observations, we found that the intraforaminal and intraspinal DRGs have a deeper position and are more completely surrounded by bone than the extraforaminal DRGs. Consequently, using the transforaminal approach alone is problematic, as it is difficult for the needle to reach intraforaminal and intraspinal DRG and therefore achieve an effective ablation fraction.

The intraspinal DRG is located close to the dural sac and due to the bony structure around the dural sac, using transforaminal and trans-lateral-zygapophysial approach is not ideal. Using a translaminar approach allows a more direct path for the needle to arrive at a more central position within the intraspinal DRG, thereby effectively improving the puncture accuracy and increasing the RFA fraction.

For the intraforaminal DRG, there is no significant difference between the ablation fraction of transforaminal approach and translaminar approach. And for extraforaminal DRG, there is also no significant difference between the ablation fraction of transforaminal and trans-lateral-zygapophysial approach. Therefore, in clinical practice, the extraforaminal DRG can be punctured using transforaminal or trans-lateral-zygapophysial approach based on the individual situation of the patient.

Use of the combined puncture approach can improve the effectiveness of RFA for all three DRG locations, particularly the intraforaminal DRG. In particular, the ablation fraction of intraforaminal DRG, with the use of combined puncture approach, was 81%, clearly higher than the transforaminal approach alone.

It is likely that transforaminal and translaminar approach tends to damage lateral regions of intravertebral DRGs, while the translaminar approach could damage the tissue on the medial part only. Using a combined puncture approach could therefore, destroy both lateral and medial DRG tissue, and a better ablation fraction can be achieved.

For the extraforaminal and intraspinal DRGs, the RF damage rate of a combined puncture approach was also significantly better. Using a combined puncture approach could increase the area of RFA, significantly increasing the damage rate.

There are some limitations to this study. Poor filling of the dural sac in some specimens may have affected the imaging results of DRG. Additionally, the number of intraforaminal and intraspinal DRGs was relatively small. Large-sample clinical studies are needed to further validate our findings.

## Conclusion

Insufficient preoperative imaging assessment and the common use of the non-specific transforaminal approach, irrespective of DRG location are the two important reasons for the poor curative effect of thoracic DRG RFA. MRI can accurately assess DRG location locations before RF surgery and 3D FIESTA scanning has an optimal visibility of thoracic DRG. Adopting tailored puncturing approaches according to different locations of DRG locations can significantly increase the DRG ablation fraction achieved.

## Methods

### MRI scan

Six normal adult corpse specimens were used as thoracic spine specimens. This study was approved by Affiliated Hospital of North Sichuan Medical College Review Board. An MRI scan was performed on each specimen. Preparation before scanning included the following: placing the spinal specimen in the head-advanced position, ligating the upper and lower ends of the spinal sac, and preserving the infusion tubes at both ends before ligation. A 10-ml syringe was used to slowly infuse 40–50 ml of water to moderately fill the spinal sac. The specimens were then scanned using the following MRI sequences: STIR T2WI, fs-FRFSE T2WI, and 3D FIESTA-c. Coronal scans ranged from T1 to L1 vertebral levels, and axial scans included the T1–12 foraminal area. Scan parameters are shown in Table 3.

**Table 3:** MRI scan parameters.

Parameters	fs-FRFSE T2WI	FSE-STIR T2WI	3D FIESTA-c
TR/TE/NEX (ms/ms/)	3000/120/2	5500/60/2	6.0/2.9/2
FOV (cm)	30–32	30–32	30–32
Thk/Sp (mm/mm)	1.6/0	4/0.5	1.0/ − 0.5
Matrix	384 × 320	384 × 320	384 × 320
Number	24–36	22–33	46–92
Time (min:s)	3:24–5:43	5:06–7:59	5:56–7:33

### Image post-processing, analysis, and data collection

The original and reconstructed images were observed in the coronal, axial, and sagittal plane. DRG identification was based on the clear observation of swelling in the spinal dorsal roots. The visibility of thoracic DRGs between multiple MRI imaging sequences were compared and with reference to previous research, the imaging visibility was divided into three levels: (1) clearly displayed DRG with sharp edges; (2) DRG with blurred shapes and unclear edges, but still identifiable; (3) DRG not clearly displayed and was unrecognizable. DRG rated as level 1 or 2 were considered visible for the purpose of determining their location. The images were observed by two imaging professors, who made a consensus on each image after consultation. The DRG location was also recorded.

### RFA

Corresponding to the different RFA puncture approaches, 14 thoracic spine specimens were divided into three groups. MRI scan was performed on each specimen to distinguish DRG position before RFA. Group A included two specimens, and RFA was performed using single transforaminal approach. Group B included six specimens, which were further divided into subgroups according to the DRG location. The subgroups included an extraforaminal subgroup, an intraforaminal subgroup, and an intraspinal subgroup. Each subgroup in Group B underwent RFA using different puncture approaches as follows. (1) Extraforaminal subgroup: If the DRG was on the right side, the transforaminal approach was used, and if on the left side, the trans-lateral-zygapophysial approach was used. (2) Intraforaminal subgroup: DRGs on the right side received the transforaminal approach, and the translaminar approach was used for DRGs on the left side. (3) Intraspinal subgroup: DRGs in the right received the transforaminal approach, and on the left side, the translaminar approach was used (Fig. 3). Finally, group C included six specimens and combined puncture approaches were used depending on the DRG position as follows. (1) Extraforaminal subgroup: the transforaminal combined with trans-lateral-zygapophysial approach was used. (2) Intraforaminal subgroup: the transforaminal combined with translaminar approach was used. (3) Intraspinal subgroup: the transforaminal combined with translaminar approach was used. Computed tomography (CT) scans were performed to confirm the RF cannula tip was located at the target position. DRGs were ablated individually using a Smith & Nephew ET-20S RF treatment system, with an ablation time of 90 s and temperature of 80 degrees Celsius.

### Specimen and pathological data collection

After RFA, the DRGs were completely removed from the thoracic spine specimen by an anatomy professor. DRGs were sectioned and hematoxylin–eosin (HE) staining was performed. The DRG sections were examined by two pathology professors under an optical microscope, and the ratio of necrotic area to healthy tissue of the entire DRG was calculated to determine the ablation fraction.

### Statistical analysis

All statistical analyses were performed using the Statistical Package for Social Sciences software (version 23.0, https://www.ibm.com/cn-zh/analytics/spss-statistics-software, IBM). Continuous variables were reported as means ± standard deviations, and categorical variables were shown as percentages. A chi-square test (χ^2^ test) was used to compare the difference in visualization of thoracic DRG with different MRI imaging sequences. For comparison of RFA fraction between groups A, B, and C, a one-way analysis of variance (ANOVA) was used. A *p* value < 0.05 was considered as significant for all statistical analyses.

### Statement

The authors confirm that all experimental protocols were approved by Affiliated Hospital of North Sichuan Medical College Review Board. The authors confirm that all methods were carried out in accordance with relevant guidelines and regulations. The authors confirm that informed consent from donor or next of kin was obtained.
